# A population pharmacokinetic model for R- and S-citalopram and desmethylcitalopram in Alzheimer’s disease patients with agitation

**DOI:** 10.1007/s10928-015-9457-6

**Published:** 2015-11-26

**Authors:** Ayman Akil, Robert R. Bies, Bruce G. Pollock, Dimitrios Avramopoulos, D. P. Devanand, Jacobo E. Mintzer, Anton P. Porsteinsson, Lon S. Schneider, Daniel Weintraub, Jerome Yesavage, David M. Shade, Constantine G. Lyketsos

**Affiliations:** Division of Clinical Pharmacology, Department of Medicine, School of Medicine, Indiana University, Indianapolis, IN USA; Campbell Institute, CAMH, University of Toronto, Toronto, ON Canada; Department of Psychiatry and Behavioral Sciences, Johns Hopkins University School of Medicine, Baltimore, MD USA; Division of Geriatric Psychiatry, New York State Psychiatric Institute and College of Physicians and Surgeons of Columbia University, New York, NY USA; Clinical Biotechnology Research Institute, Roper St. Francis Healthcare, Charleston, SC USA; University of Rochester School of Medicine and Dentistry, Rochester, NY USA; University of Southern California Keck School of Medicine, Los Angeles, CA USA; Perelman School of Medicine at the University of Pennsylvania, Philadelphia, PA USA; Stanford University School of Medicine and VA Palo Alto Health Care System, Stanford, CA USA; Johns Hopkins Bloomberg School of Public Health, Baltimore, MD USA; Johns Hopkins Bayview and Johns Hopkins School of Medicine, Baltimore, MD USA; Department of Pharmaceutical Sciences, School of Pharmacy and Pharmaceutical Sciences, State University of New York at Buffalo, Buffalo, NY 14214 USA

**Keywords:** Citalopram, Pharmacokinetics, Agitation, Alzheimer’s disease

## Abstract

The citalopram for Alzheimer’s disease trial evaluated citalopram for the management for agitation in Alzheimer’s disease patients. Sparse data was available from this elderly patient population. A nonlinear mixed effects population pharmacokinetic modeling approach was used to describe the pharmacokinetics of R- and S-citalopram and their primary metabolite (desmethylcitalopram). A structural model with 4 compartments (one compartment/compound) with linear oral absorption and elimination described the data adequately. Overall, the model showed that clearance of the R-enantiomer was slower than the clearance of the S-enantiomer. Without accounting for any patient-specific covariates, the population estimate of the metabolic clearance of citalopram was 8.6 (R-citalopram) and 14 L/h (S-citalopram). The population estimate of the clearance of desmethylcitalopram was 23.8 (R-Dcit) and 38.5 L/h (S-Dcit). Several patient-specific covariates were found to have a significant effect on the pharmacokinetics of R,S-citalopram and desmethylcitalopram. A significant difference in the metabolic clearance of R-citalopram between males and females (13 vs 9.05 L/h) was identified in this analysis. Both R- and S-citalopram metabolic clearance decreased with age. Additionally, consistent with literature reports S-citalopram metabolic clearance increased with increasing body weight and was significantly influenced by CYPC19 genotype, with a difference of 5.8 L/h between extensive/rapid and intermediate/poor metabolizers. R,S-desmethylcitalopram clearance increased with increasing body weight. This model may allow for the opportunity to delineate the effect of R- and S-citalopram on pharmacodynamics outcomes related to the management of agitation in Alzheimer’s disease.

**Electronic supplementary material:**

The online version of this article (doi:10.1007/s10928-015-9457-6) contains supplementary material, which is available to authorized users.

## Introduction

Patients with dementia, including those with Alzheimer’s disease (AD), often suffer from agitation [1]. Symptoms of agitation include restlessness, tumultuous emotions and violent/excessive movements [2]. There is no approved FDA pharmacological intervention for the management of agitation in patients with AD [3]. Nonetheless, several drug classes are used to manage agitation in AD patients including antipsychotics, anticonvulsants and antidepressants [2, 3].

A recent multi-center, randomized, placebo-controlled, double-blind, parallel group trial, The Citalopram in Alzheimer’s disease (CitAD) study, evaluated the efficacy of citalopram for the management of agitation in AD patients [4]. The dose of citalopram was set to a target of 30 mg/daily, with dose adjustment possible based on response and tolerability. Overall the study showed that patients who received citalopram improved compared with the placebo group on agitation outcomes. However, worsening of cognition and QTc prolongation was observed in the citalopram group [4, 5].

Citalopram, a selective serotonin reuptake inhibitor (SSRI), is a racemic mixture composed of 50 % (R)-(−)-citalopram and 50 % (S)-(+)-citalopram. It has been suggested that most of the antidepressant effects are attributable to S-citalopram [6, 7]. This is further supported by studies showing that S-citalopram (a.k.a., escitalopram) alone exerts better efficacy than racemic citalopram for depression [8–11]. Citalopram is readily absorbed after oral administration and has a bioavailability of approximately 80 %, a volume of distribution of 12–16 L/kg, and an elimination half-life in healthy adults of 30–35 h [12, 13]. It is metabolized by liver cytochrome P450 enzymes (CYP 2C19, 2D6 and 3A4) to its major metabolite desmethylcitalopram, which undergoes further demethylation mediated by CYP2D6, to form didemethylcitalopram [12, 13]. The systemic clearance of citalopram decreases with age due to a decrease in metabolic activity [14], with the elimination half-life approximately 30 % longer in the healthy elderly population compared to a young population [15]. Previously published population pharmacokinetic models for citalopram and S-citalopram have also shown decreased clearance of the drug with increasing age [16, 17].

Due to the sparse sampling approach utilized in most studies of elderly people it is difficult to perform a classical pharmacokinetic analysis, which requires extensive sampling. Population mixed effects pharmacokinetic models are widely used as a tool to describe pharmacokinetics and explore exposure–response relationships in drug development [18]. The advantage of a population pharmacokinetic approach is that it can leverage the sparse data available in order to make inferences about population and individual level pharmacokinetics. As the objective of the CitAD trial was to assess the efficacy and side effects of citalopram for the management of agitation in AD patients, understanding the pharmacokinetics of citalopram in this elderly patient population is key towards establishing an exposure–response relationship. This in turn is critical towards establishing the effectiveness of citalopram as a therapeutic agent for management of agitation in this patient population.

The objective of this study was to use mixed effects population pharmacokinetic modeling approach to describe the pharmacokinetics of R,S-citalopram and their primary metabolites (R,S-desmethylcitalopram) and identify patient-specific covariates that contribute to the variability in pharmacokinetics parameters. The effect of age, weight, sex and CYP2C19 genotype on the pharmacokinetics parameters of the parent and metabolite of both enantiomers were assessed using this population pharmacokinetic approach.

## Methods

### Participants data

**Fig. 1:**
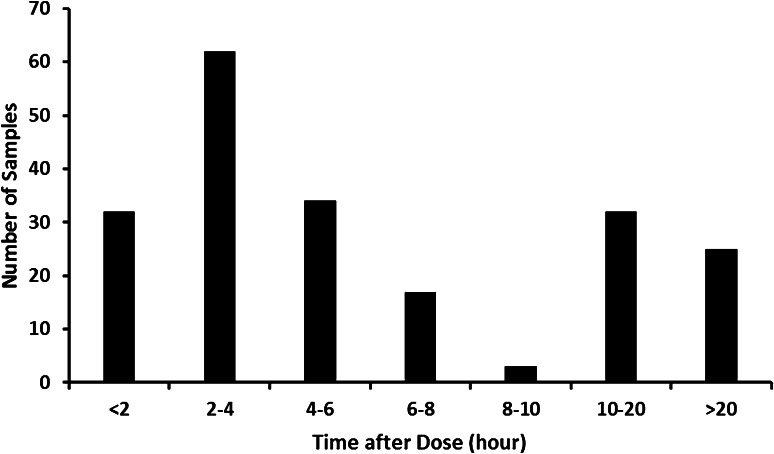
Frequency histogram of the sampling distribution for concentration measurements of the different compounds

**Fig. 2:**
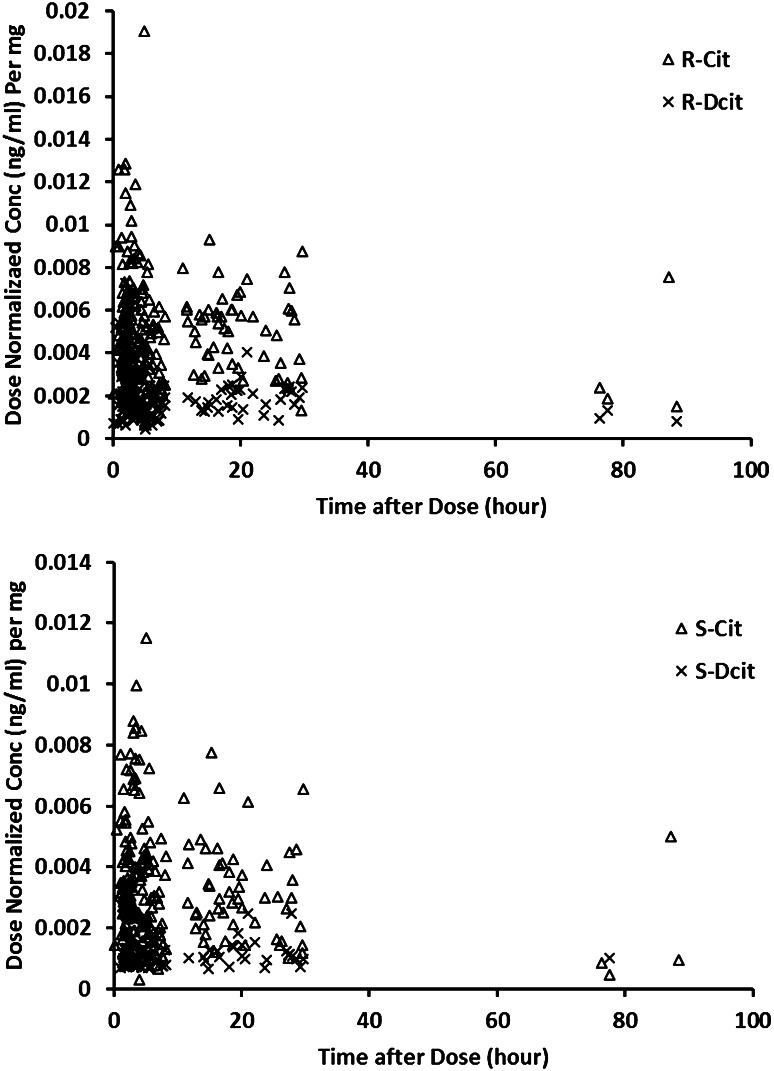
Spread of R,S-citalopram and R,S-desmethylcitalopram dose normalized plasma concentration versus time after dose

Ninety-four patients from the CitAD study received citalopram. A starting dose of 10 mg was titrated up over 2 weeks to the target of 30 mg daily, provided as a single dose in the morning of three capsules each containing 10 mg. Plasma samples were collected at weeks 3, 6, and 9. R,S-citalopram and R,S-desmethylcitalopram concentrations were determined using a sensitive high-performance liquid chromatography method (HPLC) with a chiral column with UV detection [19, 20]. Figure 1 illustrates the sampling design by showing the number of plasma samples versus time after dose. Scatter plots of concentration versus time after dose stratified per enantiomer (Fig. 2) show the spread of plasma concentrations of each compound after dose. The limit of quantitation for each enantiomer was 5 ng/mL except for S-desmethylcitalopram where the limit was 10 ng/mL. More detailed information about the CitAD trial design can be found in previously published reports [4, 5].

### Population pharmacokinetic model development

#### Base model

A step-wise approach was adopted to build a joint model for both citalopram enantiomers and their primary metabolites (desmethylcitalopram). In this approach a model was built for R-citalopram parent followed by the addition of its metabolite. Similarly a parent-metabolite model was built for S-citalopram. The two models were then combined into one model describing the pharmacokinetics of R,S-citalopram and their two primary metabolites. Nonlinear mixed effects modeling was performed using NONMEM 7.1 (ICON Software Development). The model was developed using ordinary differential equations implemented in NONMEM as (ADVAN6 TRANS1). The model equations were as follows:

1 $$\frac{dC\left( 1 \right)}{dt} = \frac{Ka \times Dose}{{V_{R} }} - \frac{{CL_{Rp} }}{{V_{R} }} \times C$$

2 $$\frac{dC\left( 2 \right)}{dt} = \frac{Ka \times Dose}{{V_{S} }} - \frac{{CL_{Sp} }}{{V_{S} }} \times C$$

3 $$\frac{dC\left( 3 \right)}{dt} = \frac{{CL_{Rp} }}{{V_{R} }} \times C\left( 1 \right) - \frac{{CL_{Rm} }}{{V_{R} }} \times C$$

4 $$\frac{dC\left( 4 \right)}{dt} = \frac{{CL_{Sp} }}{{V_{S} }} \times C\left( 2 \right) - \frac{{CL_{Sm} }}{{V_{S} }} \times C$$

where *C*(1) is the concentrations of R-citalopram, *C*(2) is the concentrations of S-citalopram, *C*(3) is the concentration of R-desmethylcitalopram and *C*(4) is the concentration of S-desmethylcitalopram. *Ka* is the absorption rate constant. *CL*_*Rp*_ and *CL*_*Sp*_ are the apparent metabolic clearance of R,S-citalopram. *CL*_*Rm*_ and *CL*_*Sm*_ are the apparent clearance of R,S-desmethylcitalopram. *V*_*R*_ and *V*_*S*_ are the apparent volume of distributions for the two enantiomers.

Based on previously published population pharmacokinetic models for Citalopram [16, 17], a one-compartment model with first order absorption and elimination was implemented for the parent compounds. A two-compartment model for the R- and S-citalopram was also tested. One and two-compartment models were then evaluated for the metabolite. Additionally, two assumptions for clearance were evaluated: either clearance allowed from parent and metabolite compartments (partial parent to metabolite conversion) or clearance allowed only from the parent to the metabolite compartment (complete parent to metabolite conversion). Direct dosing into the metabolite compartments was also evaluated. A statistical model was also included to describe the between subject variability (BSV) and residual error. BSV was assumed to be log-normally distributed. The relationship between a pharmacokinetic parameter (P) and its variance could therefore be expressed as follows:

$$P_{j} = P_{TV} * e^{\eta p}$$

where *P*_*j*_ was the value of the pharmacokinetic parameter for the jth individual, *P*_*TV*_ was the typical value of P for the population, and *η*_*p*_ denoted the difference between *P*_*j*_ and *P*_*TV*_, independently, which was identically distributed with a mean of 0 and variance of ω^2^. The residual variability was composed of but not limited to experimental errors, process noise, and/or model misspecification. This variability was modeled using additive, proportional and combined structures.

Population and individual specific parameters were determined in this analysis. Model parameters for both the base model and the final model were estimated by the first-order conditional estimation (FOCE) with interaction method.

#### Final model

The final model was developed by evaluating the effect of subject-specific covariates on pharmacokinetic parameter estimates. Both continuous covariates (age, weight, and BMI) and discrete covariates (CYP2C19 genotype, and sex) were tested.

The effect of the continuous covariates on pharmacokinetic parameter estimates was tested using a centered additive and power model. Age was centered on a value of 60 years, weight was centered on a value of 70 kg and BMI was centered on a value of 25 lbs/in^2^.

With regard to the discrete covariates, the effect of sex on pharmacokinetic parameter estimates was tested as follows:

$${\text{For sex}}:P_{TV} = \left[ {\theta_{1p} *{\text{ sex}}} \right] + [\theta_{2p} *(1 - {\text{sex}})],{\text{ sex}} = 0 \, \left( {\text{males}} \right)\quad {\text{or}}\quad 1\left( {\text{female}} \right)$$

where *θ*_1*p*_ was the corresponding parameter estimate for females and *θ*_2*p*_ was for males. CYP2C19 genotype was regrouped to have three possible values (EM/RM = 1, IM/PM = 2 and missing = 3). The effect of CYP2C19 genotype on PK parameter estimates was tested as follows:

$${\text{IF GEN}} = 1{\text{ THEN}}P_{TV} = \theta_{1p}$$

$${\text{IF GEN}} = 2{\text{ THEN}}\;P_{TV} = \theta_{2p}$$

$${\text{IF GEN}} = 3{\text{ THEN}}\;P_{TV} = \theta_{3p}$$

where *θ*_1*p*_ was the corresponding parameter estimate for EM/RM, *θ*_2*p*_ was for IM/PM, and *θ*_3*p*_ was for missing.

All covariates were incorporated into each parameter in a stepwise fashion. The covariate was retained in the model if the objective function value (OFV) decreased by 3.84. The ∆OFV is assumed to be distributed according to a Chi square distribution (χ^2^); therefore for one degree of freedom a 3.84 difference in the OFV would be significant for a *p* value level of 0.05. Goodness-of-fit plots were also used as additional criteria during model development. A nonparametric bootstrap was conducted with 100 replicates in order to obtain uncertainty in the parameter estimates.

## Results

### Patient characteristics

Patient demographics and characteristics are summarized in Table 1. Of the total 94 patients who provided concentration samples, 81 participants data was included in the population mixed effects pharmacokinetic analysis with 41 males (50.6 %) and 40 females (49.4 %). The average age of the participants was 77.8 years with an average body weight of 71.5 kg and average BMI of 26.3. Among all patients the CYP2C19 frequencies for EM, RM, IM and PM were 53.1, 3.7, 21, and 3.7 %. CYP2C19 genotypes were missing for 18.5 % of patients.

**Table 1:** Patient demographics and characteristics

Number of subjects	81
Total number of observations	
R-citalopram	205 (2.5 observation/subject)
R-desmethylcitalopram	179 (2.2 observation/subject)
S-citalopram	205 (2.5 observation/subject)
S-desmethylcitalopram	109 (1.3 observation/subject)
Sex	
Male	41 (50.6 %)
Female	40 (49.4 %)
Age, years, mean ± SD (range)	77.8 ± 8.2 (47–90)
Weight, kg, mean ± SD (range)	71.5 ± 17.2 (40–122.3)
Body mass index, lbs/in^2^, mean ± SD (range)	26.3 ± 5.2 (15.4–41.6)
CYP2C19 genotype	
Extensive metabolizers	43 (53.1 %)
Rapid metabolizers	3 (3.7 %)
Intermediate metabolizers	17 (21 %)
Poor metabolizers	3 (3.7 %)
Missing	15 (18.5 %)

### Population pharmacokinetic modeling

**Fig. 3:**
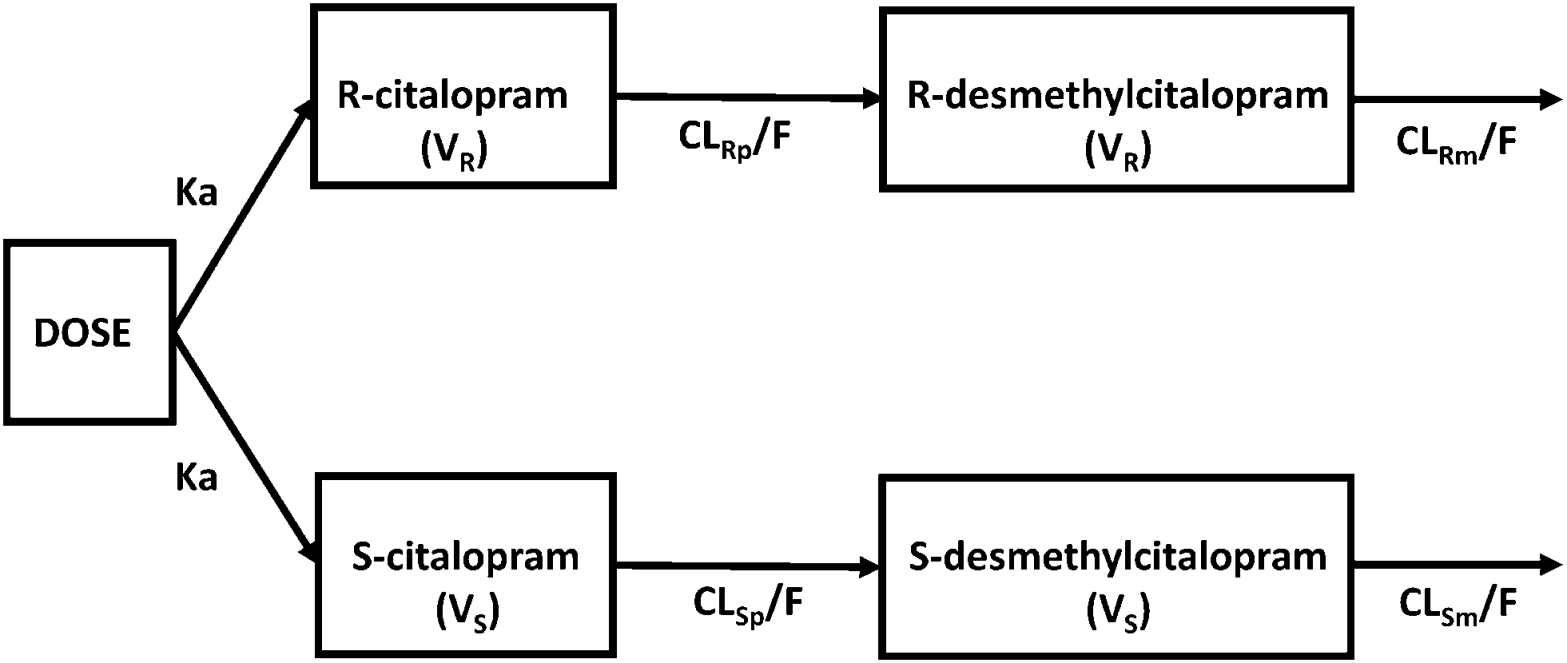
Final model compartmental structure

The structure of the final model which best described the pharmacokinetics of R,S-citalopram and their two primary metabolites (R,S-desmethylcitalopram) in this patient population consisted of four compartments (one for each compound) with dosing into the parent compartments, complete parent to metabolite conversion and linear metabolite elimination (Fig. 3). The residual error was separated with an additive structure for R-citalopram, a proportional structure for R-desmethylcitalopram and both S-citalopram and desmethylcitalopram. The oral absorption rate constant was assumed to be equal for both enantiomers. Both the parent and metabolite for the two enantiomers were assumed to have the same volume of distribution.

In the base model without any covariates added, the estimated population apparent metabolic clearance was 8.6 L/h for R-citalopram and 14 L/h for S-citalopram. R-desmethylcitalopram estimated population apparent clearance was 23.8 L/h whereas S-desmethylcitalopram estimated population apparent clearance was 38.5 L/h. The estimated population apparent volume of distribution was 2050 L for R-citalopram and 1450 L for S-citalopram.

**Fig. 4:**
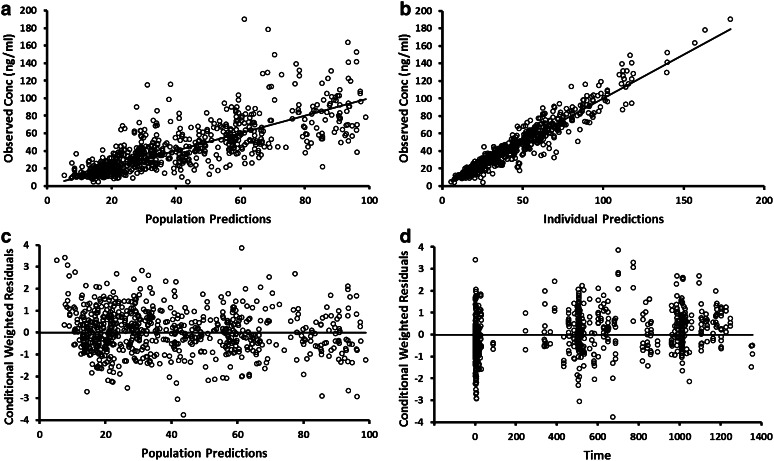
Diagnostic plots of the final pharmacokinetic model. **a** Population predicted versus observed concentrations. **b** Individual predicted versus observed concentrations. **c** Conditional weighted residuals versus concentration. **d** Conditional weighted residuals versus time

Several patient-specific covariates (age, sex, body weight, and CYP2C19 genotype) had a significant effect on different pharmacokinetic parameters. A centered power model was chosen to model the effects of continuous covariates (age and weight) on pharmacokinetic parameter estimates. Final model development steps are listed in Table 2. Diagnostic plots for the final model are shown in Fig. 4. Additionally, diagnostic plots stratified by compound are shown in supplementary Figs. 1, 2, 3, and 4 Estimates of the population pharmacokinetic parameters from the full model along with the standard errors are listed in Table 3. Uncertainty in parameter estimates was calculated by performing a nonparametric bootstrap.

**Table 2:** Covariate selection for final model

	Model	−2LL	∆ −2LL	df	*p* value^a^
Univariate forward selection					
R-enantiomer	Base	1465.08			
CL_Rp_	Added age	1449.53	−15.55	1	8.03E−5
	Added sex	1434.19	−15.34	1	8.99E−5
	Full parent model + base metabolite model	2361.97			
CL_Rm_	Added weight	2335.72	−26.25	1	3.00E−7
S-enantiomer	Base	1330.84			
L_Sp_	Added weight	1319.60	−11.24	1	8.00E−4
	Added age	1310.87	−8.73	1	0.003
	Added CYP2C19 genotype	1301.93	−8.94	2	0.01
	Full parent model + base metabolite model	1743.85			
CL_Sm_	Added weight	1721.89	−21.96	1	2.78E−6
Stepwise backward elimination					
R,S-enantiomers	Final model	4052.32			
CL_Rp_	Removed age	4070.12	17.8	1	2.5E−5
	Removed sex	4070.56	18.24	1	1.9E−5
CL_Rm_	Removed weight	4084.99	32.67	1	1.0E−8
CL_Sp_	Removed weight	4059.46	7.14	1	7.5E−3
	Removed age	4067.82	15.5	1	8.3E−5
	Removed CYP2C19 genotype	4061.49	9.17	2	0.01
CL_Sm_	Removed weight	4078.95	26.63	1	2.5E−7

^a^Calculated at 0.05 significance level

**Table 3:** Final model pharmacokinetic parameter estimates

Parameter	Final model estimate	Bootstrap, median (95 % CI)	
R-enantiomer			
CL_Rp_/F for male, L/h	13	13.8 (13.5–14.1)	R-citalopram apparent metabolic clearance
CL_Rp_/F for female, L/h	9.05	10.3 (10.1–10.5)	
V/F, L	1830	1605 (1440–2090)	R-citalopram apparent volume of distribution
Ka, h^−1^	1 (Fixed)	NA	Absorption rate constant
CL_Rm_/F, L/h	24.4	23.5 (23.1–23.7)	R-desmethylcitalopram apparent clearance
$\omega_{\text{CLp}}$, %	26.38	28.7 (27.8–30)	Variance of the BSV of R-citalopram apparent metabolic clearance
$\omega_{\text{V}}$, %	166.73	107.2 (92.6–121.2)	Variance of the BSV of R-enantiomer apparent volume of distribution
$\omega_{\text{CLm}}$, %	30.61	34.9 (34.5–35.9)	Variance of the BSV of R-desmethylcitalopram apparent clearance
σ, ng/ml (additive)	13.42	13.6 (13.3–13.9)	Variance of the residual error
σ, % (proportional)	21.54	20.7 (20.4–21.3)	
S-enantiomer			
CL_Sp_/F for EM/RM, L/h	22.1	21.9 (21.2–22.8)	S-citalopram apparent metabolic clearance
CL_Sp_/F for IM/PM, L/h	16.3	16.7 (15.9–17.2)	
CL_Sp_/F for Missing, L/h	16.8	17 (16.1–17.6)	
V/F, L	1390	1310 (1130–1420)	S-citalopram apparent volume of distribution
Ka, h^−1^	1 (Fixed)	NA	Absorption rate constant
CL_Sm_/F, L/h	38.8	38.9 (38.4–39.2)	S-desmethylcitalopram apparent clearance
$\omega_{\text{CLp}}$, %	38.34	36.7 (35.8–38.5)	Variance of the BSV of S-citalopram apparent metabolic clearance
$\omega_{\text{V}}$, %	75.37	62.3 (59.5–68.9)	Variance of the BSV of S-enantiomer apparent volume of distribution
$\omega_{\text{CLp,V}}$, %	47.1	67 (54–82.7)	Covariance of the BSV of S-citalopram apparent volume of distribution and metabolic clearance
$\omega_{\text{CLm}}$, %	20.49	20.1 (19.4–20.6)	Variance of the BSV of S-desmethylcitalopram apparent clearance
σ, % (proportional)	21.61	21.6 (21–22.1)	Variance of the residual error

**Fig. 5:**
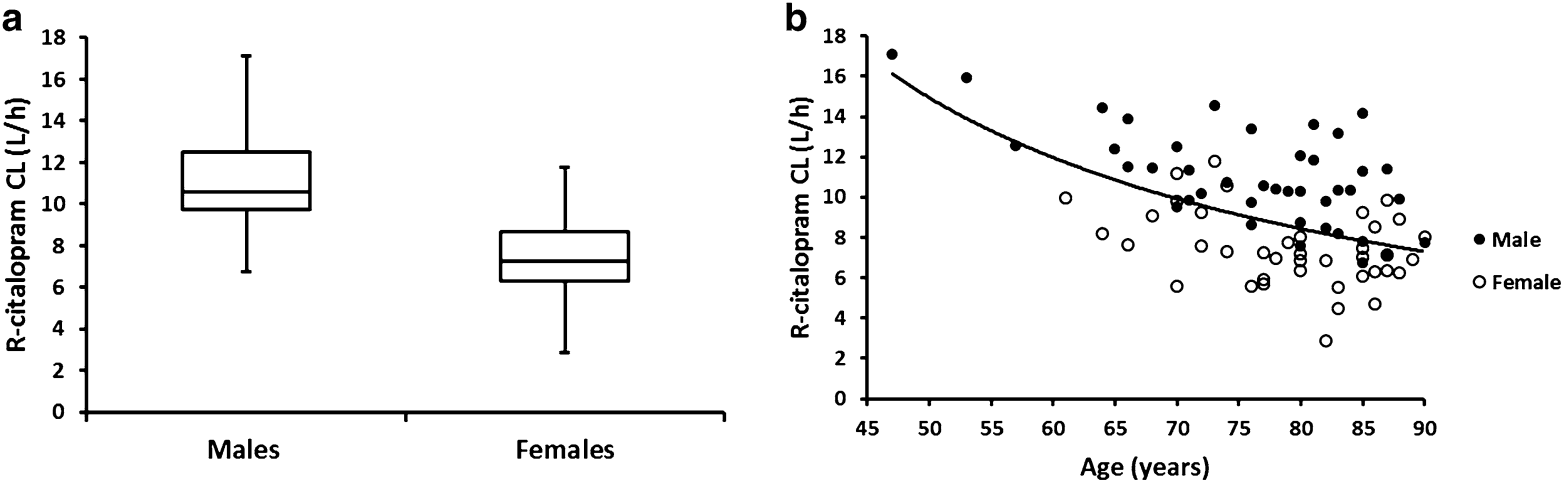
R-citalopram metabolic clearance by patient **a** sex and **b** age (*fitted line* represents the relationship of clearance with age described by a power model)

The apparent metabolic clearance of R-citalopram was approximately 30 % higher in males compared with female patients (13 L/h for males and 9.05 L/h for females). Additionally, there was a decrease in the apparent metabolic clearance of R-citalopram with increased patient age. This relationship was described in the final model by a centered power function as follows: CL_Rp_/F = CL_0_/F × (Age/60)^−0.822^. Analysis of post-processed individual empirical Bayes estimates showed that the apparent metabolic clearance for R-citalopram in subjects aged <70 years was 27.6 % faster than in subjects aged 70–79 and 43.5 % faster than in subjects aged 80–90 years. A graphical representation of the relationship between the empirical Bayes estimates of the apparent metabolic clearance of R-citalopram with patient sex and age is shown in Fig. 5. No significant effects of body weight, BMI and CYP2C19 genotype were found on the apparent metabolic clearance of R-citalopram.

**Fig. 6:**
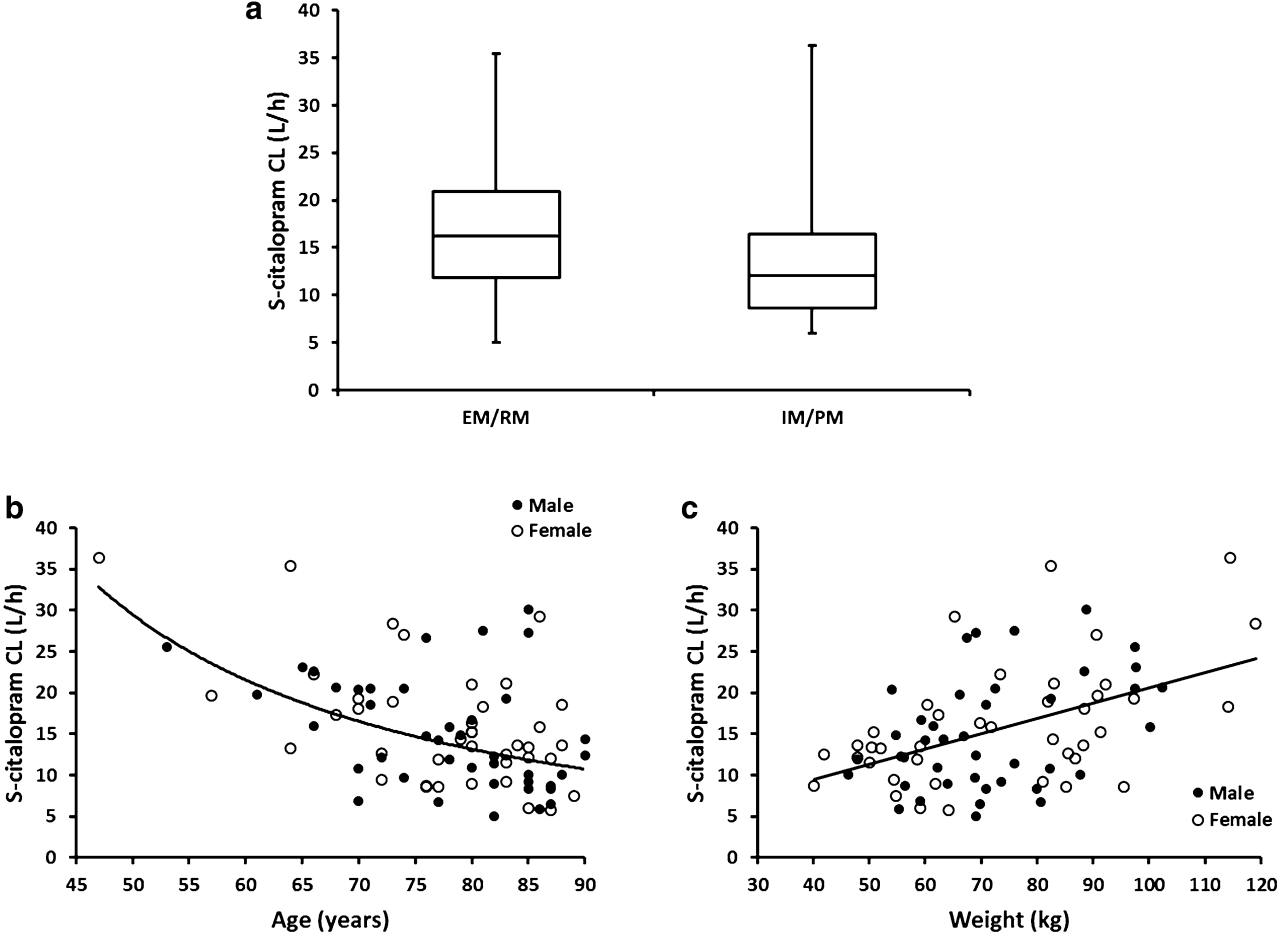
S-citalopram metabolic clearance by **a** CYP2C19 genotype, **b** age (*fitted line* represents the relationship of clearance with age described by a power model) and **c** patient body weight (*fitted line* represents the linear relationship of clearance with weight)

Similarly the apparent metabolic clearance of S-citalopram decreased with increased patient age. This was modeled using a centered power function as follows: CL_Sp_/F = CL_0_/F × (Age/60)^−1.33^. Analysis of post-processed individual empirical Bayes estimates showed that the apparent metabolic clearance for S-citalopram in subjects aged <70 years was 49.1 % faster than in subjects aged 70–79 and 65.7 % faster than in subjects aged 80–90 years. On the contrary, increased patient body weight resulted in an increase in the apparent metabolic clearance of S-citalopram. This relationship was also modeled by a centered power function as follows: CL_Sp_/F = CL_0_/F × (WT/70)^0.75^. Finally, CYP2C19 genotype was a significant factor in the apparent metabolic clearance of S-citalopram. Patients who were EM/RM had about 36 % higher apparent metabolic clearance than those who were IM/PM. The estimated population apparent metabolic clearance of S-citalopram was 22.1 L/h for EM/RM, 16.3 L/h for IM/PM and 16.6 L/h for subjects with missing CYP2C19 genotype. Figure 6 shows the graphical representation of the relationship between the apparent metabolic clearance of S-citalopram and the patient specific covariates identified to be significant.

**Fig. 7:**
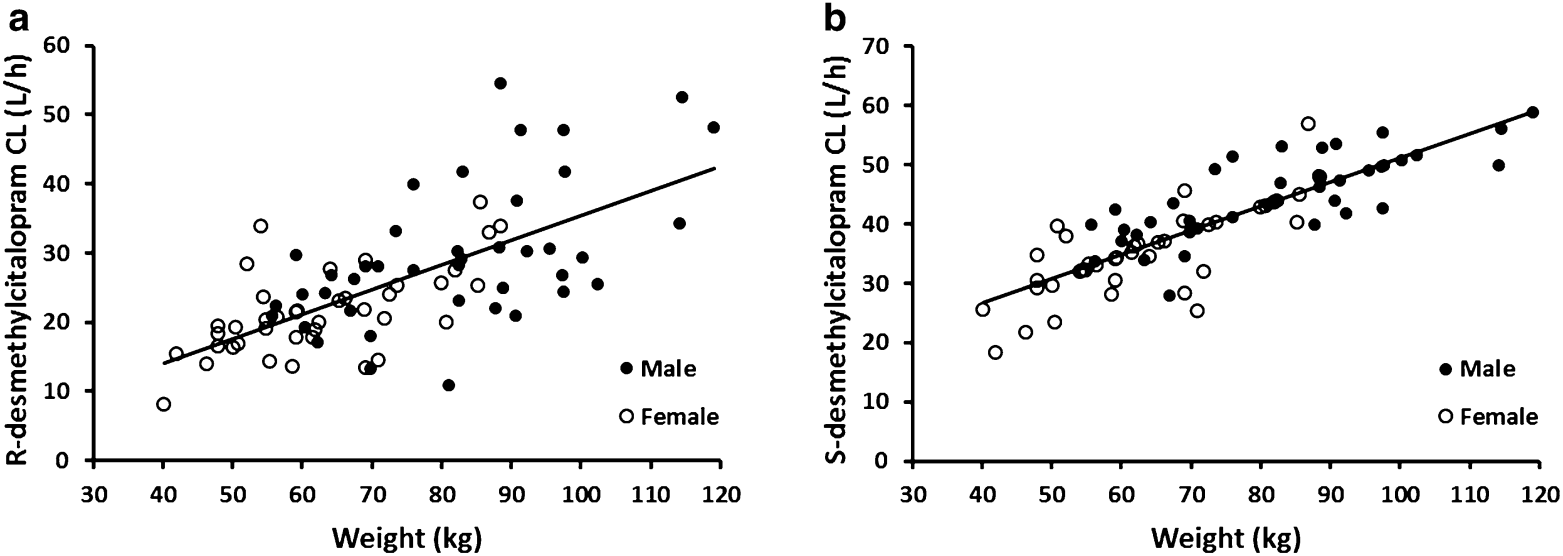
R-desmethylcitalopram (**a**) and S-desmethylcitalopram (**b**) clearance by body weight. *Fitted lines* represent the linear relationship of metabolite clearance with weight

The apparent clearance of the two metabolites R,S-desmethylcitalopram showed a significant relationship with patient body weight modeled with a centered power function as follows: CL_m_/F = CL_0_/F × (WT/70)^0.75^. Increased body weight resulted in increased apparent metabolite clearance. Post-processed individual empirical Bayes estimates showed that the average apparent clearance of R-desmethylcitalopram was 20.14 ± 5.26 L/h for patients with body weights of <70 and 29.12 ± 9.95 L/h for patients with body weights of ≥70 kg. The average apparent clearance of S-desmethylcitalopram was 34.41 ± 5.78 L/h for patients with body weights of <70 and 46.22 ± 6.68 L/h for patients with body weights of ≥70 kg. Figure 7 shows the relationship between the metabolites apparent clearance and patient body weight in a scatter plot. It is noteworthy to mention that final model estimates showed that apparent clearance of S-desmethylcitalopram (38.8 L/h) was faster than R-desmethylcitalopram (24.4 L/h).

## Discussion

In this study, using a population approach, the population pharmacokinetics of R,S-citalopram and their primary metabolites (desmethylcitalopram) were successfully captured and described in elderly Alzheimer’s disease patients who received citalopram for the treatment of agitation. This analysis revealed that patient specific covariates (body weight, age, sex and CYP2C19 genotype) contributed differentially to the variability in the pharmacokinetic parameters of the two parent and metabolite enantiomers. The findings of this analysis are in line with several published studies that described the population pharmacokinetics of citalopram [16, 17]. Additionally, the results of our analysis are consistent with those derived from studies with intensive sampling design [21] with an s-citalopram apparent volume of distribution over 1000 L and a half-life of up to 40 h (our analysis shows similar apparent volume of distribution but higher overall half-life of about 43 h in the EM/RM group in an older group of psychiatric patients). It is noteworthy to mention that, compared to previously published population PK analyses for citalopram, this population analysis goes further by delineating the pharmacokinetics for each enantiomer and its primary metabolite.

Diagnostic plots of the final model showed good fitness of the model to the observed data. The scatterplots of the observed versus predicted population concentrations and observed versus predicted individual concentrations were distributed symmetrically around the line of unity. The conditional weighted residuals were distributed symmetrically around zero. No systematic shift in residuals was evident from the plots of conditional weighted residual versus predicted population concentrations or time after dose.

Post-processed individual empirical Bayes estimates of the two enantiomers showed that the average apparent metabolic clearance of R-citalopram (8.73 ± 2.74 L/h) was significantly slower than S-citalopram (13.76 ± 6.77 L/h) (*p* < 0.05, Mann–Whitney *U* test). Tanum et al. showed that the plasma level of R-citalopram was significantly higher than S-citalopram in patients receiving racemic citalopram [22]. This difference was dose dependent and in the dose group of (20–30 mg/daily, which is the dose range in our analysis) the mean R/S ratio was 1.99. The difference in apparent metabolic clearance of the enantiomers found in our analysis may explain the observed difference in plasma concentrations observed by Tanum et al. The individual empirical Bayes estimates also showed that the average apparent clearance of the R-desmethylcitalopram (23.70 ± 9.05 L/h) was slower than S-desmethylcitalopram (39.75 ± 8.74 L/h) (*p* < 0.05, Mann–Whitney *U* test). In a clinical study, it was shown that under steady-state conditions the serum concentrations of S-desmethylcitalopram represented 42 % of the total racemic serum concentrations indicating faster clearance of the S-enantiomer which is consistent with our finding [23].

The influence of sex on the pharmacokinetics of citalopram has been controversial in the literature. Reis et al. reported that women have lower citalopram clearance and higher dose corrected citalopram plasma concentrations than men [24, 25]. On the contrary, another study showed no difference in dose corrected citalopram comparing men and women [26]. A population pharmacokinetic analysis by Bies et al. did not reveal an influence of sex on citalopram pharmacokinetics [16]. Our analysis revealed a sex effect on the apparent metabolic clearance of R-citalopram. No effect of sex was found on S-citalopram or R,S-desmethylcitalopram apparent clearance. Post-processed individual empirical Bayes estimates showed that the average apparent metabolic clearance for R-citalopram was 10.59 ± 2.41 L/h (males) and 7.25 ± 1.87 L/h (females). This finding could explain the conflicting reports in the literature with regard to the sex effect on citalopram PK. Since citalopram is administered as 50/50 racemic mixture it is difficult to capture the sex differences on each enantiomer PK without having separate measurement of the two enantiomer or by conducting a traditional PK analysis. Given the measurement of each enantiomer and by using a population level approach (accounting for fixed and random effects), our analysis provided the opportunity to understand the influence of sex on the overall citalopram PK by showing that this effect is on the R-citalopram metabolic clearance only. This observation may have implications for assessing the differential impact of the two enantiomers on clinical outcomes. Ho et al. showed that R-citalopram exposure (represented by area under the curve AUC) was mainly responsible for the QTc prolongation and negative impact on cognition (Mini-Mental State Examination score). Moreover higher R-citalopram AUC was associated with the probability of worse patient response to treatment as compared to placebo [27]. The differences in the patient specific factors affecting the R- and S-enantiomers of citalopram suggest the potential for within-individual differences in the R and S enantiomer concentrations that could contribute to different responses attributable to these enantiomers.

Model results showed a decrease in the apparent metabolic clearance of R,S-citalopram with increasing age. This finding is in agreement with previously published population models. In a population analysis of S-citalopram, the mean clearance for subjects aged 50–65 years was 21.74 L/h [17]. Our analysis showed that the apparent metabolic clearance of S-citalopram for similar age group (47–68 years) was 22.63 L/h. So even though more than 85 % of subjects in our analysis are elderly (≥70 years) our model prediction of S-citalopram apparent metabolic clearance in younger subjects was very similar with previous published findings.

In addition to age, our model revealed that the apparent metabolic clearance of S-citalopram was influenced by weight and CYP2C19 genotype. This has been captured and described previously by Jin et al. [17]. In that study EM/RM subjects were reported to have a 33.7 % faster S-citalopram clearance than IM/PM. Despite the fact that the studied population in our study is older than the one in Jin et al. study, our analysis showed that EM/RM subjects had a 25.6 % faster apparent metabolic clearance of S-citalopram than IM/PM subjects. No impact of CYP2C19 genotype was observed in our analysis on the apparent metabolic clearance of R-citalopram. In a study by Herrlin et al. the impact of CYP2C19/CYP2D6 on the metabolism of R,S-citalopram was evaluated [28]. Results showed no impact of genotype on the exposure (i.e. AUC) of R-citalopram. Other studies that have assessed the impact of CYP2C19 genotype on racemic citalopram reported significant differences in exposure or disposition of citalopram due to CYP2C19 genotype [29–31]. Our findings suggest that those observed differences may be attributed to the impact of CYP2C19 genotype on S-citalopram only.

As mentioned previously, our analysis showed that the overall apparent clearance of the R-enantiomer was slower than that of the S-enantiomer. Additionally, there was a further decline in the apparent metabolic clearance of R-citalopram in females. All leads to the assumption that exposure to R-citalopram in this patient population would be higher than S-citalopram. Post-processed individual empirical Bayes estimates showed that exposure (AUC) to R-citalopram (1.46 ± 0.58 mg/L/h) was significantly higher than that of S-citalopram (0.97 ± 0.45 mg/L/h) (*p* < 0.05, Mann–Whitney *U* test). Published studies suggested that R-citalopram antagonizes or counteracts S-citalopram activity [32–36]. Taken together, the findings of our study suggest that it may be warranted to evaluate S-citalopram (escitalopram) for treatment for the management of agitation in elderly patients with AD.

One caveat in this analysis is that both enantiomers are administered simultaneously in one pill containing the racemic citalopram. Hence we cannot determine the impact of this co-administration on the pharmacokinetics of each enantiomer. Ideally, a separate administration of the S-enantiomer would allow for a comparison with the racemic drug administration. In such case a clearer understanding of the pharmacokinetics/pharmacodynamics relationship would be feasible. That being said, there are sufficient differences within individuals in the R- and S-citalopram enantiomer disposition that they are not perfectly correlated and would allow for the exploration of enantiomer specific effects on observed responses. On the other hand, clearance was shown in this analysis to be the driver behind the difference in exposure to the R- versus S-enantiomer. It is noteworthy to mention bioavailability in this analysis was not explored as IV administration and oral of the individual enantiomers was not part of the study design and therefore the bioavailability could not be determined. Hence it is safe to assume that the observed differences in exposure of the two enantiomers may be related to underlying differences in bioavailability. There are no reports of bioavailability of R-citalopram however reported bioavailability for racemic citalopram and S-citalopram is 80 % [21]. Finally, although our model structure assumes complete parent to metabolite conversion, it is important to note that a small portion of citalopram is excreted in the urine unchanged (8–10 % in the case of S-citalopram) [21]. With more intensive sampling (i.e. collection of urine samples) the model could be refined to reflect urinary excretion.

## Conclusions

The findings of this study was able to delineate between the pharmacokinetics of R versus S-citalopram and -desmethylcitalopram in elderly patient population. We have identified a differential influence of some patient specific covariates on the pharmacokinetics of both enantiomers and their primary metabolite, all of which have implications on understanding the pharmacokinetic/pharmacodynamics relationship in order to optimize therapy and tailor it to the needs of the patient. In particular, the potential for higher exposure of R-citalopram in women may increase the possibility of untoward cognitive impacts but this will need to be studied specifically using escitalopram to confirm.

## Electronic supplementary material

Supplementary material 1 (DOCX 11 kb)
