# Supplementary material for: A population pharmacokinetic model for R- and S-citalopram and desmethylcitalopram in Alzheimer’s disease patients with agitation
Source: J Pharmacokinet Pharmacodyn. 2015 Nov 26;43:99–109. doi: 10.1007/s10928-015-9457-6 (PMC4720707; doi:10.1007/s10928-015-9457-6)
Supplement: Supplementary file 1 — Supplementary material 1 (DOCX 11 kb) [file 10928_2015_9457_MOESM1_ESM.docx]

**Supplementary Figure 1.** Diagnostic plots of the final pharmacokinetic model for R-citalopram only. (a) Population predicted versus observed concentrations. (b) Individual predicted versus observed concentrations. (c) Conditional weighted residuals versus concentration. (d) Conditional weighted residuals versus time.

**Supplementary Figure 2.** Diagnostic plots of the final pharmacokinetic model for S-citalopram only. (a) Population predicted versus observed concentrations. (b) Individual predicted versus observed concentrations. (c) Conditional weighted residuals versus concentration. (d) Conditional weighted residuals versus time.

**Supplementary Figure 3.** Diagnostic plots of the final pharmacokinetic model for R-desmethylcitalopram only. (a) Population predicted versus observed concentrations. (b) Individual predicted versus observed concentrations. (c) Conditional weighted residuals versus concentration. (d) Conditional weighted residuals versus time.

**Supplementary Figure 4.** Diagnostic plots of the final pharmacokinetic model for S-desmethylcitalopram only. (a) Population predicted versus observed concentrations. (b) Individual predicted versus observed concentrations. (c) Conditional weighted residuals versus concentration. (d) Conditional weighted residuals versus time.
